# Intracellular NAD^+^ levels are associated with LPS-induced TNF-α release in pro-inflammatory macrophages

**DOI:** 10.1042/BSR20150247

**Published:** 2016-02-29

**Authors:** Abbas Jawad Al-Shabany, Alan John Moody, Andrew David Foey, Richard Andrew Billington

**Affiliations:** * School of Biological Sciences, University of Plymouth, Drake Circus, Plymouth, PL4 8AA, U.K.; † School of Biomedical and Healthcare Sciences, University of Plymouth, Drake Circus, Plymouth, PL4 8AA, U.K.

**Keywords:** immune responses, lipopolysaccharide (LPS), macrophages, pyridine nucleotides, second messenger, TNF-α

## Abstract

Metabolism and immune responses have been shown to be closely linked and as our understanding increases, so do the intricacies of the level of linkage. NAD^+^ has previously been shown to regulate tumour necrosis factor-α (TNF-α) synthesis and TNF-α has been shown to regulate NAD^+^ homoeostasis providing a link between a pro-inflammatory response and redox status. In the present study, we have used THP-1 differentiation into pro- (M1-like) and anti- (M2-like) inflammatory macrophage subset models to investigate this link further. Pro- and anti-inflammatory macrophages showed different resting NAD^+^ levels and expression levels of NAD^+^ homoeostasis enzymes. Challenge with bacterial lipopolysaccharide, a pro-inflammatory stimulus for macrophages, caused a large, biphasic and transient increase in NAD^+^ levels in pro- but not anti-inflammatory macrophages that were correlated with TNF-α release and inhibition of certain NAD^+^ synthesis pathways blocked TNF-α release. Lipopolysaccharide stimulation also caused changes in mRNA levels of some NAD^+^ homoeostasis enzymes in M1-like cells. Surprisingly, despite M2-like cells not releasing TNF-α or changing NAD^+^ levels in response to lipopolysaccharide, they showed similar mRNA changes compared with M1-like cells. These data further strengthen the link between pro-inflammatory responses in macrophages and NAD^+^. The agonist-induced rise in NAD^+^ shows striking parallels to well-known second messengers and raises the possibility that NAD^+^ is acting in a similar manner in this model.

## Abstract (precis)

Bacterial lipopolysaccharide induces changes in intracellular NAD^+^ levels in a pro-inflammatory, but not an anti-inflammatory, macrophage model that are correlated with the release of the pro-inflammatory cytokine tumour necrosis factor-α (TNF-α).

## INTRODUCTION

Previous work has revealed a fascinating link between metabolism and the ability of an organism to mount an immune response [1]. This link is bidirectional with examples of changes in metabolism being required in order to mount the appropriate response and with immune mediators being able to modulate metabolism [2]. Indeed, many diseases that were previously considered to be pure metabolic disorders now being reconsidered also as inflammatory diseases and *vice versa* [2]. Immunometabolism has been particularly well studied in macrophages, myeloid derived phagocytes with different phenotypes that can be involved in numerous innate immune functions from bacterial killing to wound healing [3]. In particular, classically activated, pro-inflammatory (M1) macrophages are predominantly glycolytic whereas alternatively activated; anti-inflammatory (M2) macrophages rely on oxidative metabolism [4]. Forcing M1s to become oxidative, or M2s to become glycolytic, leads to a switch in phenotype. A front-line response to bacterial infection needs to be short lived as the consequent production of large amounts of pro-inflammatory mediators such as tumour necrosis factor-α (TNF-α) can lead to a pro-inflammatory cascade resulting in potentially fatal cytokine storm [5].

Glycolysis can operate under anaerobic conditions (such as those that may be found at sites of infection) for short periods of time but is unsustainable in the long term due to lactate and ROS production, reflecting the role of M1s. On the other hand, M2s are involved in much longer term anti-inflammatory processes in which cytokine storm is not possible. Thus M2s are much more suited to aerobic oxidative metabolism of glucose and fatty acids which, while slower than glycolysis, produces more ATP per gram of substrate and can be sustained practically indefinitely. Given that NAD^+^ is an absolute requirement for glycolysis, it is perhaps not surprising that NAD^+^ levels have also been linked to immune function in macrophages. Indeed, a key role has been shown for NAD^+^ in the positive control of transcription of the major M1 pro-inflammatory cytokine TNF-α via NAD^+^-dependent sirtuin deacetylase activity [6]. Decreasing NAD^+^ levels, SIRT6 knock out or inhibition, blocks transcription of TNF-α suggesting a link between the redox and thus metabolic, state of cell and pro-inflammatory responses. During glycolysis, NAD^+^ is reduced to NADH whereas NADH can be oxidized back to NAD^+^ during anaerobic lactic fermentation or during mitochondrial oxidative phosphorylation [7], thus, NAD^+^/NADH ratios provide readout of glycolytic activity that would appear to be important in the regulation of TNF-α transcription. Conversely, it has been found that TNF-α itself can modulate intracellular NAD^+^ content in macrophages, by regulating the expression of a number of NAD^+^ homoeostasis enzymes, presumably as some sort of feedback regulation [8]. Furthermore, LPS, a classic pro-inflammatory stimulus that leads to TNF-α release, has also been shown to mediate the expression of NAMPT (nicotinamide phosphoribosyl transferase, a key enzyme in the NAD^+^ recycling pathway) in activated monocytes and macrophages [8,9].

It is thus clear that NAD^+^ is involved in macrophage pro-inflammatory responses involving TNF-α. Previous interest in the pharmacological modulation of NAD^+^ homoeostasis has led to a number of drugs entering clinical trials as chemotherapeutic agents for cancer [10]. Given the link between NAD^+^ and TNF-α, it is also possible that such agents could be used to modulate pro-inflammatory responses. Indeed, FK866, an inhibitor of the NAD^+^ recycling pathway, decreased TNF-α secretion in THP-1 monocytes and human monocyte during LPS stimulation [10,11]. We have investigated whether NAD^+^ levels could also be involved in the LPS-stimulated secretion of TNF-α in monocytic THP-1 cells differentiated to generate M1-like and M2-like macrophage models. The generated subsets differed in their relative levels of expression of NAD^+^-homoeostasis enzymes and in their resting levels of NAD^+^ suggesting that NAD^+^ homoeostasis may be phenotype specific. LPS induced robust TNF-α production that was accompanied by large changes in intracellular NAD^+^ levels in M1-like cells, whereas this was not the case in M2-like cells. LPS also induced changes in the expression of NAD^+^-homoeostasis enzymes that differed between subsets. These data extend the intimate relationship between NAD^+^ levels and homoeostasis and TNF-α. Furthermore, to our knowledge, this is the first report of agonist stimulated NAD^+^ rises and bringing NAD^+^ a step closer to being classified as a second messenger.

## MATERIALS AND METHODS

### Cell culture maintenance, macrophage differentiation and stimulation

The human monocytic leukaemia cell line THP-1 (ECACC) was used routinely between passage 8 and 24 and maintained in complete growth RPMI 1640 medium (Lonza) supplemented with 10% (v/v) FBS (Sera lab), 2 mM L-glutamine, 100 unit/ml penicillin and 100 μg/ml streptomycin (P/S) (Lonza). Cells were kept at 37°C, under 5% CO_2_ in a humidified incubator.

For THP-1 differentiation into macrophage-like subsets, the cells were plated (at density of 1×10^6^ cell/ml) in complete medium and treated with PMA (M1-like; 25 ng/ml; 3 days followed by 5 days resting in fresh medium) or 1,25-(OH)_2_-Vitamin D_3_ (Sigma–Aldrich; M2-like; 10 nM; 7 days) [12].

Cells were stimulated with 100 ng/ml LPS derived from *Escherichia coli* K12-LPS (Autogen-Bioclear Ltd.) for the indicated time intervals. Dead cells were aspirated and the live cells and supernatant were harvested by centrifugation at 200 ***g*** for 5 min, and stored at −20°C until the day of analysis. For cell counting, adherent cells (M1-like) were de-attached by trypsinization using 0.5% (v/v) of trypsin with 0.2% (v/v) EDTA (Sigma–Aldrich).

### NAD^+^ cycling assay for the measurement of intracellular NAD^+^ levels

For the determination of NAD^+^ content, the washed cell pellets were lysed with 0.2 M HCl and heated to 100°C for 10 min. The intracellular NAD^+^ content were measured using a modified enzymatic cycling assay [13]. Briefly, the acid extract was mixed with 151 μl of cycling buffer: 98 mM bicine pH 8.0, containing 0.2 M sodium hydroxide, 1.63 mM PES (phenazine ethosulfate), 0.412 mM MTT (3-(4,5-Dimethyl-2-thiazolyl)-2,5-diphenyl-2H-tetrazolium bromide), 9.8% (v/v) absolute ethanol, 3.92 mM EDTA and 5 μl of yeast alcohol dehydrogenase (ADH) (400 unit/mg) were incubated for 30 min in the dark at room temperature. The absorbance was read at 565 nm using a plate reader (Versa Max, Molecular Devices).

### Quantitative analysis of cytokine levels

To quantify extracellular TNF-α levels, cell culture supernatants were analysed using sandwich ELISA according to the manufacturer's instructions. Supernatants were harvested from THP-1-derived macrophage subsets at the indicated time points, and protocols were followed and compared with standard curves between the range of 7 and 5000 pg/ml using the international standards available from NIBSC, Potter's Bar, UK. Briefly, 96-well ELISA plates (Nunc, Fisher Scientific) were coated with (4 μg/ml) of mouse anti-human TNF-α (BD Pharmingen), in PBS as a coating buffer, and incubated overnight at 4°C.

Thereafter, samples were added into the plate, and incubated overnight at 4°C. For TNF-α detection, biotinylated mouse anti-TNF-α at 1 μg/ml in 2% BSA (w/v in PBS) buffer were applied, and horseradish peroxidase streptavidin conjugate were added, followed by incubation with TMB reagent (Insight Biotechnology Ltd.). Colorimetric development was assayed spectrophotometrically by an OPTI Max tuneable microplate reader set to 450 nm and analysed by Soft max Pro version 2.4.1 software (Versa Max, Molecular Devices).

### RNA extraction, reverse transcription and qPCR

Total RNA was extracted from approximately 1×10^6^ cultured cells, and isolated using Gen Elute Mammalian Total RNA kit (Sigma–Aldrich) with a DNase I Digestion treatment for 15 min according the manufacturer's instructions. The amount and purity of RNA was measured using a Nano-drop Plus spectrophotometer (Bioscience, Cambridge Bioscience Ltd). Total RNA (25 ng/ml) was subsequently reverse transcribed in 20 μl total volume containing: 2.5 μM random nonamers, 10 unit/μl RT and 500 μM deoxynucleotide mixture, for 1 h at 42°C before quantitative PCR (qPCR) was performed. The primers used for qPCR are listed in Table 1.

**Table 1:** Primer sequences used for qPCR

Gene	Sequence (5′ ➔ 3′)	Size (bp)	Gene	Sequence (5′ ➔ 3′)	Size
GAPDH	For: CCCACTCCTCCACCTTTGAC	20	IDO	For: GCCTGCGGGAAGCTTATG	18
	Rev: CTGTTGCTGTAGCCAAATTCGT	22		Rev: TGGCTTGCAGGAATCAGGAT	20
CD38	For: GCACCACCAAGCGCTTTC	18	NMNAT	For: TCATTCAATCCCATCACCAACA	22
	Rev: TCCCATACACTTTGGCAGTCTACA	24		Rev: AGGAGAGATGATGCCTTTGACAA	23
CD157	For: GGGAAGGCAGCATGAAAGTC	20	NAMPT	For: TCCGGCCCGAGATGAAT	17
	Rev: GGTCCACGCACTGTAAGAGCTT	22		Rev: TGCTTGTGTTGGGTGGATATTG	22

The qPCR was performed to quantify specific mRNA using SYBR green detection kit according to the manufacturer's instructions (Sigma–Aldrich). The cDNA product was utilized in the reaction mixture with 0.4 μM of specific forward/reverse primer sets alongside with (0.025 unit/μl reaction) of SYBR-Green Jumpstart Tag ready mixture (Sigma–Aldrich). The reactions were performed under the following conditions: 94°C for 2 min followed by 40 cycles of 94°C for 30 s, 60°C for 30 s and 72°C for 30 s. The quality of the RNA samples was confirmed by 1.5% (w/v) agarose gel electrophoresis. The relative amounts of mRNA, for each target transcript, were normalized according to the house-keeping gene (GAPDH) glyceraldehyde-3-phosphate dehydrogenase [14].

### Statistical analysis

Statistical calculations were performed using Statgraphics Centurion XVI (Stat Point Technologies Inc.). All data were tested for normality using the Anderson–Darling test (*P*<0.05 was used as the cut off for non-normal data). Levene's test was used to check for differences in variance between each group. For normally distributed data, one-way ANOVA followed by Tukey's test was performed. Data are reported as means±S.E.M. for triplicate samples from three independent experiments unless otherwise indicated. Differences were considered significant if *P*<0.05.

## RESULTS

**Figure 1:**
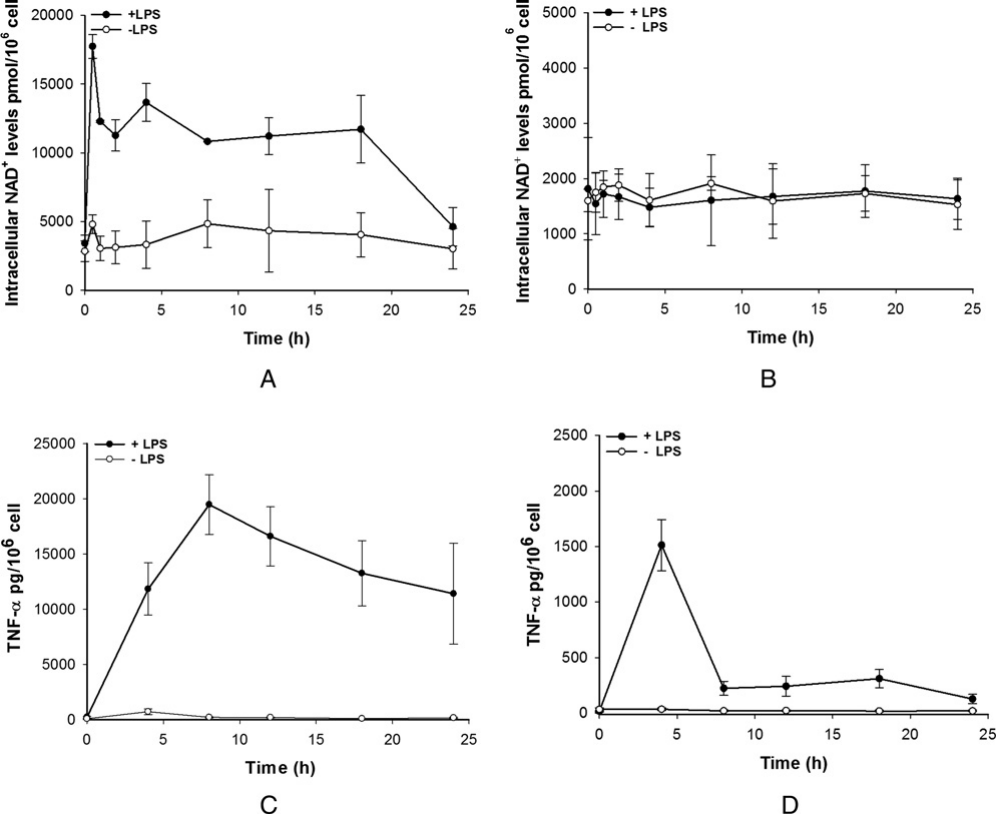
Intracellular NAD^+^ and TNF-α levels in THP-1-derived macrophages subsets LPS effect on NAD^+^ levels in pro-inflammatory (**A**; M1-like) and anti-inflammatory (**B**; M2-like) macrophages. TNF-α levels in M1-like (**C**) and M2-like (**D**) macrophages after LPS challenge. Data shown are mean±S.E.M. for three separate experiments (*n*=3).

The link between NAD^+^ and TNF-α is now well established in macrophages. Briefly, NAD^+^ regulates production of TNF-α in a sirtuin-dependent manner [6] and TNF-α has been shown to modulate the expression of a number of enzymes that are involved in NAD^+^ homoeostasis [8]. In order to investigate this link further, intracellular NAD^+^ level changes have been investigated during LPS stimulation of THP-1 monocytic cell line-derived macrophages. This system has been shown to generate cells that express markers and are functionally very close to pro- (M1) or anti- (M2) inflammatory macrophages [12,15]. Due to the heterogeneity of macrophage function, distinct M1-like pro-inflammatory and M2-like anti-inflammatory phenotypes were investigated to elucidate any potential differences. Upon LPS stimulation, M1-like cells showed a distinctive change in NAD^+^ levels that consisted of an initial fast increase, peaking at 30 min followed by a lower but sustained high level of NAD^+^ that lasted for several hours, starting to return to unstimulated levels at 24 h post-stimulation. Unstimulated cells showed a constant low level of NAD^+^ throughout the experiment (Figure 1A). In contrast, LPS stimulation of M2-like cells failed to increase intracellular NAD^+^ levels and both stimulated and unstimulated cells showed similar levels of NAD^+^ throughout the experiment. Basal levels of NAD^+^ in unstimulated M1-like and M2-like cells were different with M1-like cells appearing to have three-fold higher level of NAD^+^ than M2-like cells (Figures 1A and 1B). During stimulation experiments, media was collected and secreted TNF-α assayed over the same time course. As expected, M1-like cells showed significant TNF-α secretion that peaked at 8 h and decayed back to basal levels by 24 h (Figure 1C). In contrast, M2-like cells secreted a relatively small amount of TNF-α which corresponded to less than 10% of that secreted by M1-like cells (Figure 1D). In M2-like cells, the TNF-α peak was short lived and had returned almost to basal levels by 8 h however there was a low level of TNF-α that persisted to beyond 18 h.

**Figure 2:**
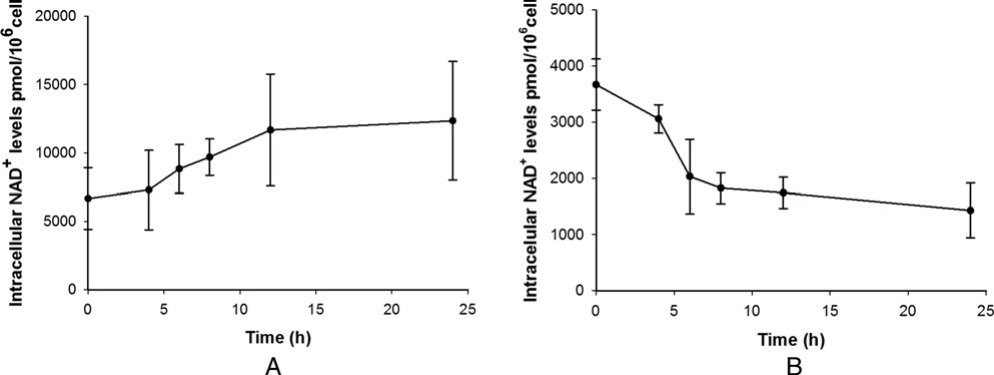
Effect of FK866 (100 nM) on NAD^+^ levels in M1-like (**A**) and M2-like (**B**) macrophages Data shown are mean±S.E.M. for three separate experiments (*n*=3).

**Figure 3:**
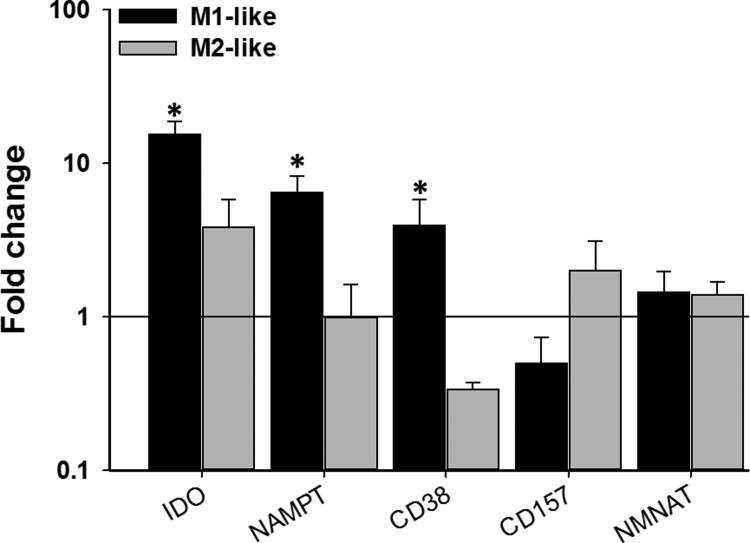
Gene expression profiles of NAD^+^ homoeostasis enzymes in M1- and M2-like macrophages relative to THP-1 monocytes Data shown are mean±S.E.M. of fold change for three separate experiments (*n*=3–5). The data were analysed by one-way ANOVA. *P<0.05.

These results suggest that LPS affects NAD^+^ homoeostasis differently in the two phenotypes. While conducting experiments to assess the effect of the NAD^+^-recycling (NAMPT) inhibitor FK866 in the two phenotypes in the absence of LPS stimulation, we noticed opposing effects on NAD^+^ levels. In M2-like cells, FK866 reduced the levels of NAD^+^ over time as would be expected due to inhibition of NAD^+^ synthesis and constitutive NAD^+^ consumption (Figure 2B). In M1-like cells, on the other hand, FK866 gradually increased NAD^+^ levels over time (Figure 2A). We are unsure as to how FK866 can cause an increase in NAD^+^ levels but this does suggest that there is a fundamental difference in how the two phenotypes perform NAD^+^ homoeostasis. To check whether the two phenotypes show different expression of some key regulators of NAD^+^ homoeostasis, real-time qPCR (RT-qPCR) was performed on unstimulated M1-like and M2-like cells and compared with expression in the monocytic progenitor THP-1 cell line (Figure 3). Both phenotypes showed increased expression of IDO compared with THP-1. The NADase CD38, that mediates NAD^+^ degradation and generates cyclic ADP ribose/NAADP [16], was significantly up-regulated in M1-like and down-regulated in M2-like cells whereas the opposite was true for CD157, a homologue of CD38 [17]. Finally, NMNAT (nicotinamide mononucleotide adenylyl transferase) that catalyses the final step in NAD^+^ biosynthesis [18], was slightly, but not significantly up-regulated in both phenotypes whereas NAMPT was significantly up-regulated only in M1-like cells.

**Figure 4:**
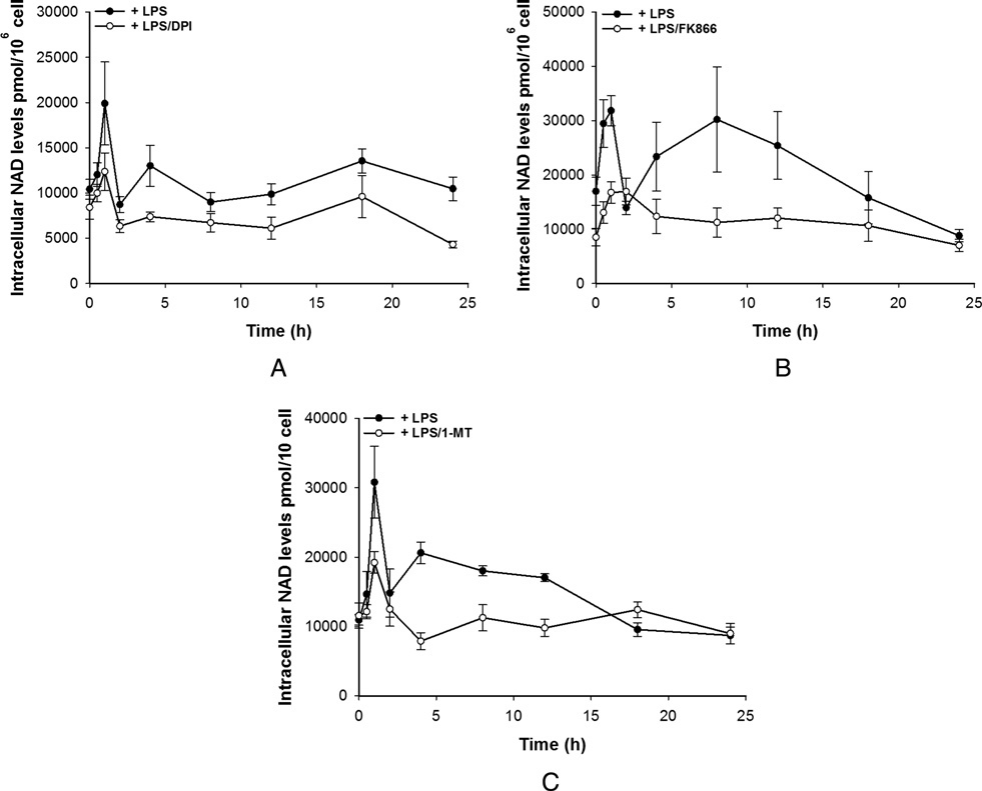
Pharmacological modulation of intracellular NAD^+^ levels by DPI (**A**), FK866 (**B**) and 1-MT (**C**) in M1-like cells Data shown are mean±S.E.M. for three separate experiments (*n*=3–4).

**Figure 5:**
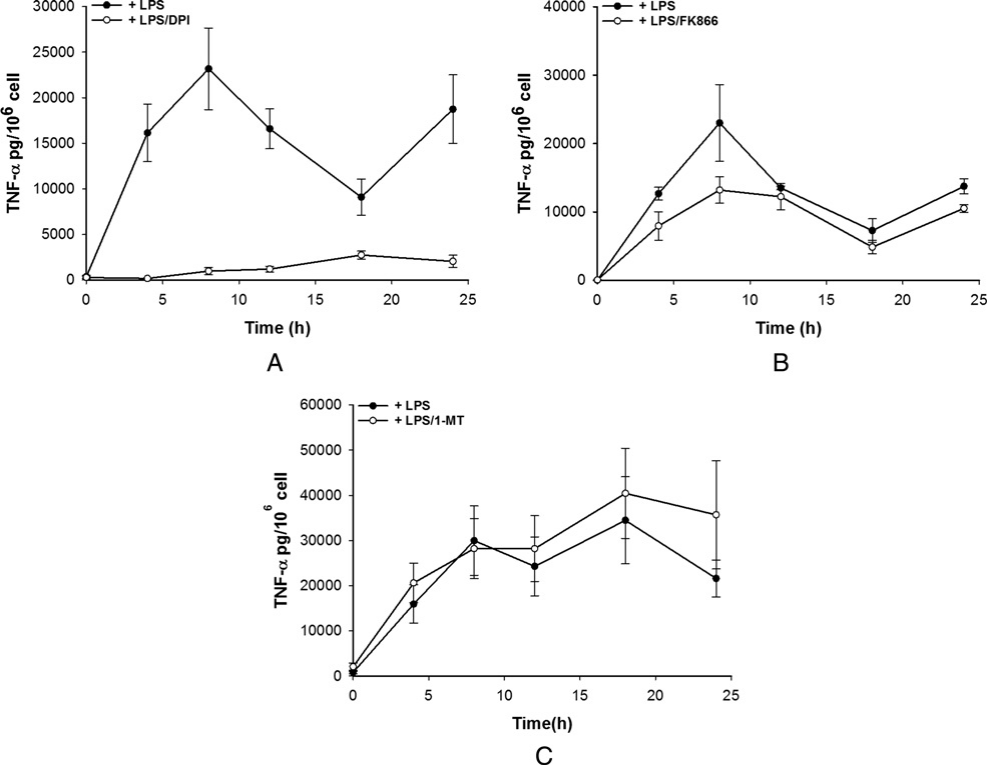
Pharmacological modulation of TNF-α release by DPI (**A**), FK866 (**B**) and 1-MT (**C**) in M1-like cells Data shown are mean±S.E.M. for three separate experiments (*n*=4).

To further investigate this and the source of the LPS-induced rise in NAD^+^ in M1-like cells, LPS stimulation of M1-like cells was performed in the presence of diphenyliodonium (DPI), an NAD(P)H oxidase (flavoenzyme) inhibitor, FK866 a NAMPT inhibitor or 1-MT, an IDO inhibitor. Concurrently TNF-α secretion was measured. In M1s, DPI was able to significantly attenuate both the initial NAD^+^ peak and the sustained rise caused by LPS suggesting that one source of the NAD^+^ is the oxidation of NADH during the respiratory burst as might be expected (Figure 4A). Surprisingly, both FK866 and 1-MT were also able to attenuate both the initial peak and the sustained rise in NAD^+^ perhaps suggesting that multiple pathways lead to LPS-induced NAD^+^ level rises (Figures 4B and 4C). Most striking was the effect of the inhibitors on TNF-α production. DPI was able to almost completely abolish TNF-α secretion although a small amount was secreted over 8–24 h but significantly less than in the absence of DPI (Figure 5A). FK866 was able to partially attenuate TNF-α secretion whereas 1-MT had no effect (Figures 5B and 5C). These data suggest that the link between NAD^+^ and TNF-α secretion is perhaps complex and may require both NAD^+^ and the activity of NAD(P)H oxidase.

**Figure 6:**
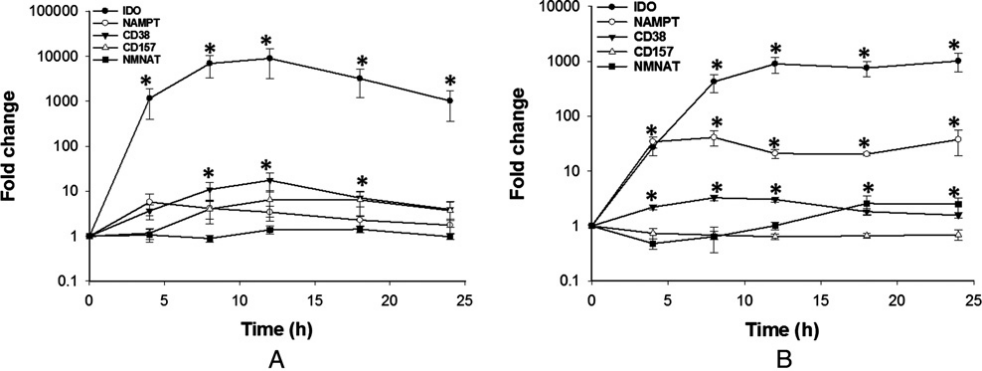
Gene expression profile of NAD^+^ homoeostasis enzymes in M1-like (**A**) and M2-like (**B**) macrophages after LPS challenge Gene expression is expressed as fold change relative to the control. Data shown are mean±S.E.M. for three separate experiments (*n*=4). The data were analysed by one-way ANOVA. *P<0.05.

Finally, it was assessed whether LPS stimulation and the concurrent changes in NAD^+^ levels and TNF-α secretion were able to feedback and affect the expression of some key regulators of NAD^+^ homoeostasis over 24 h. In M1-like cells, LPS stimulation caused a significant increase in IDO mRNA with smaller increases in CD38, CD157 and NAMPT expression whereas NMNAT expression was unaffected (Figure 6A). The increase was only significant for CD38. Despite LPS not being able to stimulate NAD^+^ changes in M2-like cells, LPS stimulation caused a similar but significant increase in IDO and in NAMPT expression (Figure 6B). CD38, CD157 and NMNAT expression were largely unaffected although there was a modest but significant increase in NMNAT and CD38 expression.

## DISCUSSION

It is becoming clear that cellular metabolic status is intrinsically linked to immune system function, particularly in macrophages [4]. One of the best studied links is that between NAD^+^/NADH and TNF-α with NAD(H) levels known to regulate TNF-α synthesis [6] and TNF-α levels known to regulate expression of NAD(H) homoeostasis enzymes [8]. In the present study, we have further extended the knowledge on this link by showing that TNF-α release is also correlated to NAD^+^ levels in a pro-inflammatory (M1-like) macrophage model. Our data show that LPS stimulation of M1-like cells induces a biphasic rise in NAD^+^ levels. LPS is well known to activate NAD(P)H oxidase [19], in a Toll-like receptor 4 (TLR4)-dependent manner (oxidative burst) which catalyses the oxidation of NAD(P)H to NAD^+^ [20]. This may explain, at least in part, the rapid peak that was observed in NAD^+^ levels as this was partially inhibited by the NAD(P)H oxidase inhibitor DPI. However, both FK866 (an NAD^+^ recycling inhibitor) and 1-MT (an IDO inhibitor) were also able to partially inhibit this initial rise. This may suggest that the NAD^+^ is also derived from a number of different synthesis and recycling pathways. The sustained rise in NAD^+^ levels also suggests that NAD^+^ synthesis occurs as it would be expected that the cell would return the redox balance after the oxidative burst relatively rapidly so as to maintain the NAD^+^/NADH ratio. Thus, it would appear that LPS has a direct effect on NAD^+^ levels in M1-like cells. LPS was unable to alter NAD^+^ levels in M2-like cells. NAD^+^ levels also appear to be correlated with secretion of TNF-α. In M1-like cells, LPS induced a robust secretion of TNF-α which was almost completely blocked by DPI and partially inhibited by FK866 suggesting that NAD^+^ levels are, in some way, related to TNF-α secretion. The exact mechanism is unclear but is has been shown that TNF-α secretion requires active glycolysis [21]. Glycolysis is inhibited by high NADH levels as NAD^+^ is absolutely required as a coenzyme. Both oxidation of NADH to NAD^+^ and a simple increase in NAD^+^ synthesis could both provide these conditions. Indeed, lactic fermentation exists to oxidize NADH to NAD^+^ under anaerobic conditions such that glycolysis may continue. M2-like cells responded to LPS by releasing a relatively small amount of TNF-α. Crucially, this was not associated with any changes in NAD^+^ levels, suggesting that the signalling between TLR4 and TNF-α release is via a different pathway compared with M1-like cells. TLR4 is known to signal via different intracellular pathways, one of which is NAD(P)H oxidase dependent which causes the oxidative burst and ROS production [22] and converts NADH to NAD^+^ [20].

One interesting outcome from our data is that the two different phenotypes appear to have quite different mechanisms and levels of machinery for NAD^+^ homoeostasis, raising the exciting possibility that NAD^+^ homoeostasis may be phenotype-specific and even an underlying cause of phenotypic differences. Indeed, previous work has shown that NAD^+^ and TNF-α are inextricably linked suggesting that high NAD^+^ may be ‘pro-inflammatory’ [6,8,23]. We found that resting NAD^+^ levels were higher in M1-like than M2-like cells and this may be linked with the finding that M1s are predominantly glycolytic [24] and thus, require relatively more NAD^+^. M1-like cells, in resting state, displayed higher expression of the recycling pathway enzymes NAMPT than both M2-like cells and the parent THP-1 cells as well as higher levels of the NADase CD38. The CD38 homologue and NADase CD157, however, displayed higher expression in M2-like cells, the converse of CD38. NAMPT is the rate limiting enzyme in the NAD^+^ recycling pathway [25], this pathway is the most active of the three known pathways that lead to NAD^+^ synthesis; the others being the *de novo* (IDO) pathway [26] and the nicotinamide riboside kinase (NRK) pathway [27]. The greatest increase in expression, compared with parent THP-1 cells, was in IDO in both phenotypes and it might be expected that the *de novo* pathway would, thus, be the biggest contributor to NAD^+^ levels. However, this pathway is known to contribute little to the intracellular NAD^+^ pools with as little as 1 in 60 molecules of NAD^+^ being derived from tryptophan [28]. As NAD^+^ levels represent a steady state between synthesis and degradation, it is perhaps surprising that the NADase, CD38, was also up-regulated in M1-like cells. This may reflect greater turnover of NAD^+^ in these cells or that the increase in CD38 is related to its receptor functions. CD38 is unusual in that it can act as both a cell surface receptor (binding to adhesion molecule-1, CD31) and as an enzyme [29] but our knowledge of how exactly the two functions interact is scant [30]. CD38 is known to be a marker of classically activated (M1-like) macrophages [31] and our results suggest that CD38 might contribute to differentiated macrophage responses. For the M2-like cells, aside from modest increases in IDO and CD157 expression, NAD^+^ homoeostasis enzymes were largely unchanged compared with the parent THP-1 cells.

Finally the gene expression of the same enzymes was investigated after LPS challenge. In M1-like cells, mRNA expression of all enzymes except NMNAT increased over 20 h after LPS addition with the largest effects on IDO and CD38. Iqbal and Zaidi [8] have shown that TNF-α application to macrophages increased the expression of the same enzymes although they also showed an increase in NMNAT expression. Furthermore, Fedele et al. [29] also showed that LPS increases CD38 expression in monocyte-derived dendritic cells in an NF-κB-dependent manner. It is likely that the TNF-α released during the experiment is partially responsible for the expression observed and thus why the results largely follow the same trend. However, the data presented here were generated after LPS stimulation and early signalling events may have initiated expression pathways that were then augmented or modulated by autocrine/paracrine signals upon TNF-α secretion. M2-like cells, that secreted a lesser amount of TNF-α, showed subtle differences in expression profiles of the enzymes. Whereas IDO and CD38 both increased, CD157 remained unchanged. Most striking was the differential regulation of NMNAT and NAMPT mRNA expression. NAMPT expression increased whereas NMNAT expression remained unchanged. Large increases in NAMPT in M1 would suggest that the cells are priming themselves for NAD^+^ production as this is the rate limiting enzyme of the recycling pathway. Although we have been unable to measure long-term effects after LPS stimulation due to limitations in the cell model, it is tempting to postulate that changes in NAD^+^ homoeostasis caused by these changes in gene expression, might contribute to changes in cell phenotype post-infection.

These data serve to reinforce the link between NAD^+^, TNF-α and inflammatory responses. Recent focus has shifted to the links between metabolism and innate immune responses and NAD^+^ is a key player both as readout of the metabolic status of a cell and as a signalling molecule. Of particular interest is that the two phenotypes appear to have different mechanisms for handling NAD^+^ and this may reflect different needs in terms of NAD^+^. Furthermore, their basal and stimulated expression profiles of a number of NAD^+^ homoeostasis enzymes also differ suggesting that they can further model how NAD^+^ homoeostasis is carried out depending on the circumstances of the cell. Although NAD^+^ is central to the links between metabolism and immunology, there has been precious little work on NAD^+^ homoeostasis in this context and this may prove to be an interesting avenue to pursue in different aspects of immune responses.

Finally, that LPS induces rises in NAD^+^ levels, suggests that NAD^+^ may be acting in a similar manner to a classical second messenger. Indeed, of the five criteria suggested by Sutherland [32], we have shown NAD^+^ fulfilling four in this work namely: agonist stimulated changes in NAD^+^ levels, NAD^+^ synthesis and degradation, antagonism of NAD^+^ action blocking the cellular response and intracellular NAD^+^ targets. This cell/response system is not ideal for intracellular application of NAD^+^ as cell damage may release TNF-α making any observations hard to interpret but should intracellular NAD^+^ application be shown to mimic agonist responses in another system, NAD^+^ could be regarded as a true second messenger, albeit a slightly unusual one.

## Abbreviations

DPI: diphenyliodonium
NAMPT: nicotinamide phosphoribosyl transferase
NMNAT: nicotinamide mononucleotide adenylyl transferase
qPCR: quantitative PCR
TLR4: Toll-like receptor 4
TNF-α: tumour necrosis factor-α
